# Genomic Signatures of Influenza A Pandemic (H1N1) 2009 Virus

**DOI:** 10.3201/eid1512.090845

**Published:** 2009-12

**Authors:** Guang-Wu Chen, Shin-Ru Shih

**Affiliations:** Chang Gung University, Taoyuan, Taiwan, Republic of China

**Keywords:** Influenza, mutation, signatures, species specificity, viruses, pandemic (H1N1) 2009 virus, research, expedited

## Abstract

Adaptive mutations that have contributed to the emergence of influenza A pandemic (H1N1) 2009 virus, which can replicate and transmit among humans, remain unknown. We conducted a large-scale scanning of influenza protein sequences and identified amino acid–conserving positions that are specific to host species, called signatures. Of 47 signatures that separate avian viruses from human viruses by their nonglycoproteins, 8 were human-like in the pandemic (H1N1) 2009 virus. Close examination of their amino acid residues in the recent ancestral swine viruses of pandemic (H1N1) 2009 virus showed that 7 had already transitioned to human-like residues and only PA 356 retained an avian-like K; in pandemic (H1N1) 2009 virus, this residue changed into a human-like R. Signatures that separate swine viruses from human viruses were also present. Continuous monitoring of these signatures in nonhuman species will help with influenza surveillance and with evaluation of the likelihood of further adaptation to humans.

## Abstract 2

Amino acid signatures at host species–specific positions may affect virus transformation.

A recent outbreak of pandemic (H1N1) 2009, previously known as the swine-origin influenza A, has infected >296,000 persons worldwide; 3,486 deaths have been reported (*1*). An increased number of infected humans can potentially alter virulence in the human population. The genomic sequences of many of the new strains of pandemic (H1N1) 2009 virus have revealed important information for promoting medical diagnosis, drug-resistance monitoring, clinical and basic research, and vaccine development. Nevertheless, analyzing adaptive mutation of the new pandemic (H1N1) 2009 virus is a priority so that researchers can evaluate the likelihood that viruses from other nonhuman species will further adapt to humans.

Pandemic (H1N1) 2009 virus consists of multiple reassorted virus genes from different origins. Of its 8 segmented genomic RNAs, 2 polymerase genes, PB2 and PA, were from the avian virus of North American lineage and were introduced into swine populations around 1998. The other polymerase gene, PB1, also evolved recently from a human seasonal influenza (H3N2) virus around the same year. This particular H3N2 PB1 gene is known to have originated from an avian virus that entered humans in 1968. However, hemagglutinin (HA), nucleoprotein (NP), and nonstructural (NS) protein genes of pandemic (H1N1) 2009 virus descended directly from the classic swine influenza A virus of North American lineage, which can be traced back to the 1918 virus. Originating from the Eurasian swine virus, the remaining 2 genes, neuraminidase (NA) and matrix (M), were introduced from birds around 1979 (*2*,*3*). Limited information is available as to how this unique combination of gene segments evolved from 1998 until it was identified in April 2009 or on the molecular transitions or evolutionary path of this virus before it was transmitted among humans.

Our previous study developed an entropy-based computational scheme to identify host-specific genomic signatures of human and avian influenza viruses (*4*). This method is based on an entropy threshold computed from the amino acid composition at the well known PB2-627 position of avian influenza viruses (entropy value of 0.4 was based on 95 avian influenza genomes, as of early 2006), which contains mostly glutamic acid in the native avian hosts of the viruses. This threshold was then used to identify the 52 species-associated positions at which each of the 2 viruses settles as a distinct amino acid residue that is characteristic of the host. Although the origin of the gene segments in pandemic (H1N1) 2009 virus has been determined (*2*,*3*), the mechanism of transformation of the host-specific amino acid signatures is unclear, because the new viral genes evolved after they were introduced into the swine population some years ago.

By adopting the entropy profiling approach, this study attempts to update influenza A viral signatures on the basis of all influenza sequences from the National Center for Biotechnology Information (NCBI). In addition to providing an updated list of human-avian signatures, this study also computes the human-swine signatures and analyzes the amino acid sequences of pandemic (H1N1) 2009 virus at the host species–specific positions to elucidate the adaptive mutation of influenza A viruses in these host species. As more new influenza virus isolates are collected and their sequences analyzed, the signatures at the host species–specific positions serve as predictors of adaptive mutation, subsequently providing valuable information to help in preparing for potential pandemics.

## Materials and Methods

### Influenza Virus Sequences

All influenza A virus protein sequences from the NCBI, as of May 28, 2009, were downloaded and analyzed. These full-length or partial sequences were grouped according to the hosts from which the viruses were isolated: humans, avian, and swine. In particular, to observe how these viruses vary in terms of residues, the newly deposited pandemic (H1N1) 2009 virus sequences were considered separately from the human isolates. For each host-specific group, sequences belonging to each viral protein were aligned using the program ClustalW (*5*). Based on the proposed signature identification procedure, 2 surface proteins, HA and NA, were not analyzed because their extensive genetic diversity prevents satisfactory multiple alignment within either human or avian viruses. As an alternatively translated protein product from the PB1 gene, PB1-F2 is also not included in the analysis because it terminates prematurely at position 12. For each of the 4 groups of data, i.e., human, avian, swine, and pandemic (H1N1) 2009, eight alignments were analyzed: PB2, PB1, PA, NP, M1, M2, NS1, and NS2. The total number of sequences varied from gene to gene and from host to host, subject to their availability at the NCBI. For human-isolated viruses (excluding strains of pandemic (H1N1) 2009 virus), >3,000 sequences of the 8 proteins were analyzed. For avian-isolated, swine-isolated, and pandemic (H1N1) 2009 viruses, the numbers of sequences were ≈3,500, 350, and 70, respectively.

### Recent Ancestors of Pandemic (H1N1) 2009 Viruses

Smith et al. (*6*) performed evolutionary analysis of the early development of the pandemic, indicating that sporadic infection of humans with triple reassortant and other subsequent reassortant swine viruses occurred before the 2009 human outbreak. To elucidate the transition of amino acid residues along this evolutionary course, we collected and analyzed the protein sequences of 18 recent ancestral swine viruses of the new H1N1 viruses (hereinafter termed “recent ancestral swine viruses”) for 1999–2009 from the ancestral lineages of the new pandemic (H1N1) 2009 strains. The sampling was based on the phylogenetic trees published in a study by Smith et al. (*6*). Although a number of swine virus origins have been reported, resulting in various genetic lineages and subtypes, we are most interested in identifying a swine virus population from which the current pandemic (H1N1) 2009 virus might have evolved directly. Not only are those 18 strains chronologically closer (after the years 1997–1998) to the pandemic (H1N1) 2009 viruses but their PB2 and PA genes are also descendants of avian viruses, which complies with the conclusion drawn from recent publications. The Appendix Table, summarizes the strain names and accession numbers of recent ancestral swine viruses included in this study.

### Entropy-based Signature Identification

For each amino acid position of the aligned sequences of the same virus type, i.e., avian, human, swine, or pandemic (H1N1) 2009, an entropy value was computed by using the formula –Σ*P_i_* × ln(*P_i_*), as described by Chen et al. (*4*). This formula follows the definition of Shannon entropy (*7*) that has been used to evaluate the diversity of a system. In this study, an entropy was used to measure the variability of aligned amino acid residues at a given genomic position, where *i* = 1 to 20 represents 20 different amino acid residues, and *P_i_* represents the probability density of the respective residue. An entropy value ranges from 0 (only 1 residue present at that position) to 2.996 (all 20 residues are equally represented). As is assumed, a position at which the entropy is less than or equal to a prespecified threshold has a consensus residue for that virus type. When viruses isolated from 2 host species are compared, a species-specific signature position is considered to have different consensus amino acid residue from each of the 2 viruses at the same position. In this study, an entropy threshold of 0.33 was used, based on the PB2-627 position of 3,391 avian influenza sequences.

## Results

In 2006, we reported 52 avian-human signatures based on a small set of influenza sequence data of 15,785 protein sequences. The selection was based on an entropy threshold value of 0.4 set at position 627 in the PB2 gene (82 Es and 13 Ks from 95 avian PB2 sequences) because that position has been considered associated with host-restriction (*8**–**11*). Of the 52 positions, 45 are in the genes PB2, PB1, PA, NP, M1, M2, NS1, and NS2 examined in this work. Today, >100,000 influenza protein sequences are available at NCBI, and a new entropy threshold of 0.33 was set based on the currently available avian sequences of PB2-627, which contain 3,113 Es, 228 Ks, 46 Vs, 2 As, and 2 Gs. This threshold was adopted to update the list of 47 avian-human signatures in Table 1 for the 8 proteins of interest. Consistent with our earlier findings, most signatures are located on the NP gene (15 positions), followed in number by PA (10 positions), PB2 (9 positions), M2 (5 positions), M1 (3 positions), PB1 (2 positions), NS2 (2 positions), and NS1 (1 position). The 20 signatures associated with PB1, NP, and M1 do not differ between the 2 datasets of 2006 and 2009. In PB2, two new signatures are identified at positions 567 and 702. These 2 positions were only just omitted from the 2006 list because their entropy values (0.490 and 0.404, respectively) exceeded the 0.4 threshold, based on the 95 avian sequences examined at that time. New entries in Table 1 also include PA-100, M2-93, and NS1-81. In 2006, although PB2-674 was reported as a signature, it is disqualified here because the entropy of 0.3376 (3,146 As, 88 Es, 87 Ts, 38 Ss, 13 Gs, 4 Vs, and 1 K for avian virus) exceeds the new 0.33 threshold at this position. Similarly, PA-382 (2,421 Ds, 311 Es, 2 Vs, and 1 N in human viruses, with an entropy of 0.3633) and NS2-70 (2,898 Ss, 352 Gs, 28 Rs, and 1 D in avian viruses, with an entropy of 0.3483) were both removed from the 2006 list.

**Table 1:** Amino acid residues of pandemic (H1N1) 2009 virus strains at 47 positions where avian–human signatures are located*

Gene	Position	Avian virus residue	Human virus residue	Pandemic (H1N1) 2009 virus residue
PB2	44	**A**(2,838), S(39), T(1)	**S**(2,734), A(30), L(2)	**A**(61)
	199	**A**(2,816), S(22), D(4), T(2), V(1)	**S**(2,781), A(8)	**A**(61)
	271	**T**(2,758), I(47), A(21), M(5), Q(1)	**A**(2,770), T(15), S(1)	**A**(61)
	475	**L**(3,355), M(25), I(1)	**M**(2,747), L(8), I(1)	**L**(61)
	567	**D**(3,116), E(257), N(18), V(3), G(3), A(2), K(1)	**N**(2,736), D(18), S(1)	**D**(61)
	588	**A**(3,175), T(91), I(72), V(43), S(3), P(1), D(1)	**I**(2,734), A(8), V(6),T(4), L(1), S(1)	**T**(61)
	613	**V**(3,343), A(29), I(19)	**T**(2,651), I(71), A(23), V(9), S(1)	**V**(61)
	627	**E**(3,113), K(228), V(46), A(2), G(2)	**K**(2,746), E(6), R(3)	**E**(61)
	702	**K**(3,232), R(131), Q(1)	**R**(2,731), K(22), G(1), I(1)	**K**(61)
PB1	327	**R**(3,340), K(54), G(1)	**K**(2,489), R(275)	**R**(80)
	336	**V**(3,350), I(26), A(16)	**I**(2,595), V(168), T(1)	**I**(80)
PA	28	**P**(2,915), S(7), L(5), T(1)	**L**(2,736), P(19), R(2), S(2), Q(1)	**P**(61)
	55	**D**(2,906), N(29)	**N**(2,752), D(13)	**D**(61)
	57	**R**(2,849), Q(77), K(4), W(3), L(2)	**Q**(2,736), R(20), L(6), K(3)	**R**(61)
	100	**V**(2,759), A(109), I(68), F(1)	**A**(2,727), V(27), T(7), I(2), S(1)	**V**(61)
	225	**S** (2,854), N(7), C(6), G(1)	**C**(2,736), S(29), G(1)	**S**(61)
	268	**L**(3,317), F(14), I(2), V(1)	**I**(2,724), L(35), V(2)	**L**(61)
	356	**K**(3,309), R(34), N(7), E(1), I (1)	**R**(2,705), K(30)	**R**(61)
	404	**A**(3,098), S(220), T(10), P(4), R(1), V(1)	**S**(2,706), A(28), P(1)	**A**(61)
	409	**S**(3,100), N(191), G(4), I(1), R(1), K(1)	**N**(2,723), S(11), I(1)	**N**(61)
	552	**T**(3,304), A(1), N(1)	**S**(2,721), T(10), N(2), R(1), I(1)	**T**(61)
NP	16	**G**(3,379), S(58), D(8), C(1)	**D**(2,884), G(16)	**G**(120), D(1)
	33	**V**(3,173), I(284), A(1), D(1)	**I**(2,876), V(25)	**I**(121)
	61	**I**(3,419), M(30), V(19), L(6)	**L**(2,881), I(19)	**I**(121)
	100	**R**(3,422), K(34), V(23), S(1)	**V**(2,842), I(52), R(4), A(3), L(1), M(1)	**V**(68), I(46)
	109	**I**(3,407), V(48), T(22), M(2), S(1)	**V**(2,820), I(77), A(3), T(3)	**I**(114)
	214	**R**(3,282), K(52), T(3), L(1)	**K**(2,897), R(25)	**R**(114)
	283	**L**(3,309), F(4), P(3), I(3)	**P**(3,062), L(19), S(3)	**L**(114)
	293	**R**(3,275), K(40)	**K**(3,020), R(65)	**R**(114)
	305	**R**(3,238), K(32), S(2)	**K**(3,052), R(33)	**K**(114)
	313	**F**(3,191), L(43), S(10), Y(1), C(1), I(1)	**Y**(3,064), F(21)	**V**(114)
	357	**Q**(2,766), K(33), T(3), R(2)	**K**(3,052), R(46), Q(5)	**K**(114)
	372	**E**(2,742), D(69), G(3), K(2)	**D**(3,051),E(51), N(1)	**E**(114)
	422	**R**(2,818), K(2)	**K**(2,891), R(51)	**R**(114)
	442	**T**(2,793), S(12), A(5)	**A**(2,890), T(51), R(1)	**T**(114)
	455	**D**(2,792), N(3), E(1)	**E**(2,890), D(51), T(1)	**D**(114)
M1	115	**V**(3,794), I(15), G(2), L(2), M(1)	**I**(3,586), V(19)	**V**(151)
	121	**T**(3,684), A(126), P(4)	**A**(3,599), T(7)	**T**(151)
	137	**T**(3,806), D(12), A(8), P(1), S(1)	**A**(3,577), T(25)	**T**(146)
M2	11	**T**(2,890), I(190), S(8), E(1)	**I**(3,805),T(102)	**T**(55)
	20	**S**(3,032),N(76), K(12), R(3), I(2)	**N**(3,859), S(49)	**S**(55)
	57	**Y**(3,040), H(5), N(1), C(1)	**H**(3,804),Y(65), D(25), Q(5), R(5), N(4)	**Y**(55)
	86	**V**(2,894), A(6), I(4), D(1), L(1), F(1), S(1)	**A**(3,781), V(26), T(10), D(1)	**V**(55)
	93	**N**(2,710), T(13), D(3), H(3), S(3), Y(2), I(1)	**S**(3,699), N(69), Q(2), R(1), H(1), I(1)	**N**(55)
NS1	81	**I**(2,652), V(43), T(8), M(2), S(1), Y(1), G(1)	**M**(2,860), I(59), V(4)	**I**(93)
	227	**E**(3,080), G(60), K(31), S(1)	**R**(2,863), G(8), K(2), W(1), E(1)	Delete
NS2	107	**L**(3,147), P(2), S(2), F(1), Q(1)	**F**(2,850), L(45), S(1), V(1)	**L**(93)

*Boldface indicates dominant amino acid residue type. PB, polymerase B; PA, polymerase A; NP, nucleoprotein; M, matrix; NS nonstructural.

Taubenberger et al. (*12*) identified 10 polymerase gene positions that separate avian viruses from human influenza A viruses. Table 1 shows 8 of them (PB2 199, 475, 567, 627, and 702; PA 55, 100, and 552), suggesting that the method is robust in finding these signatures. Two other polymerase gene positions that Taubenberger et al. also reported are PB1-375 and PA-382; the latter has already been mentioned above. The other missing position in Table 1, PB1-375, has an entropy value of 0.8865 for human and 0.6338 for avian viruses. This position was also excluded from the 2006 list because of an entropy of 0.698 from avian viruses, which substantially exceeded the 0.4 threshold.

To elucidate the potential adaptive mutations of the pandemic (H1N1) 2009 viruses, we studied the amino acid sequences of pandemic (H1N1) 2009 viruses at the positions that represent the so-called species-specific signatures of avian and human viruses. As shown in the last column of Table 1, 36 of the 47 positions display avian-like signatures in the pandemic (H1N1) 2009 virus. Two positions, PB2-588 and NP-313, exhibit neither avian- nor human-like signatures. Eight human-like signatures were found in pandemic (H1N1) 2009 strains, except for NS1-227, in which all new viruses have an early-terminating NS1 protein and, therefore, contain no residue.

Table 1 presents the updated avian-human signatures for influenza A viruses; Table 2 summarizes the swine-human signatures. Medical literature documents that the swine virus population has distinct evolutionary lineages that originated from the classic 1918 virus referred to as classic or North American swine virus, and the others of post-1979 Eurasian swine virus and subsequent triple reassortants. Because the residue diversity at many positions markedly increased for these swine viruses because of their distinct origins, only 8 swine-human signatures met the 0.33 threshold. Unlike some positions in which human-like signatures of pandemic (H1N1) 2009 were found (Table 1), in this study, all 8 locations of the swine-human signature of this new virus are characteristic of swine. Notably, Table 1 lists all 8 positions in Table 2, with each having the same signature as in the avian virus. Restated, avian and swine viruses contain the same amino acid residue at the 8 human-swine signature positions.

**Table 2:** Amino acid residues of pandemic (H1N1) 2009 virus strains at 8 positions where swine–human signatures are located*

Gene	Position	Swine virus residue (all subtypes)	Human virus residue	Pandemic (H1N1) 2009 virus residue
PB2	44	**A**(301), S(27), C(1)	**S**(2,734), A(30), L(2)	**A**(61)
PA	268	**L**(325), I(31), T(1)	**I**(2,724), L(35), V(2)	**L**(61)
	552	**T**(280), S(25)	**S**(2,721), T(10), N(2), I(1), R(1)	**T**(61)
M1	137	**T**(429), A(39)	**A**(3,577), T(25)	**T**(146)
M2	57	**Y**(343), H(23), R(2)	**H**(3,804),Y(65), D(25), Q(5), R(5), N(4)	**Y**(55)
	86	**V**(324), A(24), S(1)	**A**(3,781), V(26), T(10), D(1)	**V**(55)
	93	**N**(320), S(23)	**S**(3,699), N(69), Q(2), R(1), H(1), I(1)	**N**(55)
NS2	107	**L**(299), F(25)	**F**(2,850), L(45), S(1), V(1)	**L**(93)

*Boldface indicates dominant amino acid residue type. PB, polymerase B; PA, polymerase A; M, matrix; NS, nonstructural.

We attempted to further elucidate the transition of the amino acid residue on the pandemic (H1N1) 2009 virus that have human signatures by sampling 18 recent ancestral swine viruses (Appendix Table). Doing so enables us to examine more closely the prevalence of amino acid residues specifically with pandemic (H1N1) 2009 viruses. Table 3 summarizes the amino acid statistics of these recent ancestral swine viruses together with avian, human, and pandemic (H1N1) 2009 sequences at the 8 positions containing human residues for pandemic (H1N1) 2009 virus in Table 1. Consider PB2-271, for example, avian viruses have signature T, whereas human viruses have signature A. Although pandemic (H1N1) 2009 viruses also have the human signature A, their predecessors, i.e., the recent ancestral swine viruses, have already acquired the human signature A at this position. PB1-336, along with PA-409, NP-33, -100, -305, and -357, follows the same residue transition, all showing human-characteristic residues in both recent ancestral swine and pandemic (H1N1) 2009 viruses. PA-356 is the only exception, where the residue in recent ancestral swine viruses still maintains an avian-characteristic K before changing to a human residue R in pandemic (H1N1) 2009 viruses. Of particular interest is whether the transition from K to R at position PA-356 is responsible for the ability of pandemic (H1N1) 2009 viruses to replicate and transmit efficiently in humans.

**Table 3:** Position-specific residue transitioning for influenza A virus among avian, recent ancestral swine, pandemic (H1N1) 2009, and human strains, for those 8 positions of pandemic (H1N1) 2009 virus showing human-characteristic signatures*

Gene	Position	Avian virus residue	Recent swine viruses residue†	Pandemic (H1N1) 2009 virus residue	Human virus residue
PB2	271	**T**(2,758), I(47), A(21), M(5), Q(1)	**A**(17), S(1)	**A**(61)	**A**(2,770), T(15), S(1)
PB1	336	**V**(3,350), I(26), A(16)	**I**(16)	**I**(80)	**I**(2,595), V(168), T(1)
PA	**356**	**K**(3,309), R(34), N(7), E(1), I(1)	**K**(16), R(1)	**R**(61)	**R**(2,705), K(30)
	409	**S**(3,100), N(191), G(4), I(1), R(1), K(1)	**N**(17)	**N**(61)	**N**(2,723), S(11),I (1)
NP	33	**V**(3,173), I(284), A(1), D(1)	**I**(18)	**I**(121)	**I**(2,876), V(25)
	100	**R**(3,422), K(34), V(23), S(1)	**V**(17), I(1)	**V**(68), I(46)	**V**(2,842), I(52), R(4), A(3), L(1), M(1)
	305	**R**(3,238), K(32), S(2)	**K**(18)	**K**(114)	**K**(3,052), R(33)
	357	**Q**(2,766), K(33), T(3), R(2)	**K**(17), R(1)	**K**(114)	**K**(3,052), R(46), Q(5)

*Boldface indicates dominant amino acid residue type. PB, polymerase B; PA, polymerase A; NP, nucleoprotein. †Eighteen recent ancestral swine viruses as listed in the Appendix Table. We consider recent ancestral strains phylogenetically neighboring to the pandemic 2009 strains, in particular for PB2 and PA genes they are clustered together with recent avian strains because the pandemic (H1N1) viruses were reported to originate from avian viruses around 1998. Note that for 1 strain, A/swine/Missouri/4296424/06(H2N3), the PA sequence was not found anywhere near the other 17 recent swine strains of interest. Two PB1 sequences, A/swine/Hong Kong/78/2003(H1N2) and A/swine/Korea/C13/2008(H5N2), were also found distantly located from the other 16 recent swine PB1 sequences. We excluded these 3 sequences from the amino acid statistics in this table because of their genetic deviation from the remaining ancestral swine viruses we have collected here.

After all 8 human residue-containing positions of pandemic (H1N1) 2009 viruses were found to be within PB2, PB1, PA, and NP protein genes, all amino acid positions of these 4 proteins were scanned for their residue transitions among the 4 virus populations shown in Table 3. The change in the amino acid that may be associated with the transformation of pandemic (H1N1) 2009 virus is summarized in Table 4. As well as PA-356, already shown in Table 2, two additional positions, PB2-684 and PA-204, showed the same dominant amino acid residue in avian and recent ancestral swine viruses, but a different dominant residue in pandemic (H1N1) 2009 viruses and human viruses. Dominance is defined here as 1 residue containing the largest sequence count compared with other residues at a particular aligned position. The previously used entropy measurement in Tables 1, 2, and 3 does not apply to the positions listed in Table 4, in which we emphasize the amino acid transition of dominant residues instead of highly conserved ones subject to the prescribed entropy threshold 0.33. Other than those 3 positions, PB1-216 was found to contain a human residue G in 8 of 9 recent ancestral swine viruses that are closer to pandemic (H1N1) 2009 viruses in the phylogenetic tree published in a study by Smith et al. (*6*). However, for the other 7 recent ancestors that are more distant from pandemic (H1N1) 2009 viruses, PB1-216 maintains an avian-residue S in 6 of 7 viruses. Our results show that the position-specific transition may serve as a molecular marker for monitoring such adaptive mutations in the future.

**Table 4:** A list of amino acid positions containing the same residue in avian and recent ancestral swine viruses, yet changed to a different one in pandemic (H1N1) 2009 and human viruses*

Gene	Position	Avian virus residue	Recent swine viruses residue†	Pandemic (H1N1) 2009 virus residue	Human virus residue
PB2	684	**A**(3,278), T(70), S(11), V(9), G(6), D(1), E(1)	**A**(18)	**S**(61)	**S**(1,944), A(806), G(2), P(2), Y(1)
PB1	216	**S**(3,299), G(62), N(37), C(11), I(7)	G(9), **S**(7)‡	**G**(80)	**G**(1,708), S(1,039), N(5), I(4), D(1)
PA	204	**R**(2,202), K(674), E(1), G(1), S(1)	**R**(17)	**K**(61)	**K**(1,776), R(991)
	**356**	**K**(3,309), R(34), N(7), E(1), I(1)	**K**(16), R(1)	**R**(61)	**R**(2,705), K(30)

*Boldface indicates dominant amino acid residue type. PB, polymerase B; PA, polymerase A. †Same 18 recent ancestral swine viruses used in Table 3. ‡PB1-216 is dominated by residue G (G[8), S[1]) when considering only a subset of 9 PB1 sequences that are phylogenetically closer to pandemic (H1N1) 2009 virus. This statistic clearly shows the amino acid residue transition from avian to human signature within the population of recent swine viruses.

## Discussion

Although most studies confer that the death rate associated with pandemic (H1N1) 2009 infection is more moderate than that of subtype H5N1 infection, its virulence may vary with adaptive mutations in viral genes, subsequently increasing the likelihood that the new virus alters its virulence in the new host species. Many of the previously identified virulence factors are apparently not involved. For instance, no E to K mutation at position 627 of PB2 is observed, which has been considered an important factor for avian virus to efficiently replicate in mammalian systems (*8**–**11*). Previous studies have indicated that PB1-F2 contributes viral pathogenesis in the mammalian system (*13*,*14*). No PB1-F2, however, is predicted in pandemic (H1N1) 2009 viruses because it terminates prematurely at position 12. Its NS1 protein is truncated at position 220 and, therefore, lacks a PDZ ligand interacting domain. As suggested recently, the presence of this PDZ ligand domain increases the pathogenicity of avian influenza A viruses (*15*). Regardless of whether these known factors are missed, a previous study has demonstrated that the virulence of pandemic (H1N1) 2009 virus is higher than that of seasonal influenza A viruses (*16*). Although a virulence marker and a host range factor may not be necessarily linked tightly, recent investigations have also demonstrated that altering PB2-627 from E to K in the avian viruses increases its virulence in the mammalian experimental system (*9**–**11*). For example, avian influenza virus subtype H7N7 reportedly infects humans (*17*). A human isolate from a fatal case had its PB2-627 changed from avian-characteristic E to K. Correspondingly, the species-associated signatures identified in this study may serve as potential molecular targets for further evaluating how they impact the virulence of pandemic (H1N1) 2009 viruses in humans.

As shown in Tables 1 and 2, the number of signature positions decreases significantly from 47 (human vs. avian) to 8 (human vs. swine), and the positions of the latter are a subset of those of the former. These observations may have the following implications. First, the 3 host species of interest differ, with each providing a unique environment for infection by the influenza virus. When the avian virus enters humans or swine, its genetic feature is shaped by a particular evolutionary path. The viruses, therefore, have different signatures. Second, some avian-like signatures are preserved in swine viruses, suggesting that both avian species and swine may provide similar conditions for harboring influenza A viruses. The body temperature may be a determinant. As is generally known, many avian species have a body temperature exceeding 40^o^C; for most pigs it is variable but still higher than the human body temperature, which is 37^o^C. Consequently, the signatures are retained when an avian virus enters the swine population, with similar signature-related viral replication mechanisms in both species. Third, the 39 signature positions shown in Table 1, but absent from Table 2, may be correlated with certain functional domains that interact with host factors unique in humans while differing significantly from those of avian and swine. Finally, the number of signature positions of swine versus humans is substantially lower than those of avian versus humans, suggesting that the species barrier to humans is easier for a swine virus to cross than for an avian virus.

The entropy-based computation depends strongly on a good multiple sequence alignment. The 2 surface proteins HA and NA are excluded from this analysis because both contain sequences that diverged sufficiently from so many subtypes of a given species. Locating conserved residues at particular positions on the basis of these alignments is extremely difficult. The entropy threshold is the other parameter requiring attention to locate a signature position. In this study, the entropy determined from PB2-627 of the aligned residues of all avian viruses is used because PB2-627 is the most laboratory-proved host-restriction marker (*8**–**11*). A complete new set of signatures can be reproduced rapidly by using a different entropy threshold based on other factors. The diverse genetic origins of influenza viruses would also have great impact on the reported signatures. The proposed entropy-based method to reach the 8 positions listed in Table 2 was based on all swine viruses of different origins, including North American-(classic 1918) origin strains, Eurasian (post-1979 avian)-origin strains, and recent triple reassortants. A comparison of, for example, all human viruses versus classic 1918-origin swine viruses before 1978 (≈75 strains, or 20% of our swine sequence population) would report 60 signature positions (data not shown). In this work, we included all swine viruses of multiple origins in producing Table 2 to consider only host-specific genomic signatures that have been shaped by the same swine species regardless of origin. For the same reason, we did not subdivide avian or human populations into lineages when reporting avian-human signatures in Table 1.

This study analyzed a complete collection of species-specific influenza A viral sequences, including the long-evolving avian, recent ancestral swine and human viruses, as well as pandemic (H1N1) 2009 viruses, which is still in its infancy. The amino acid sequence transition of pandemic (H1N1) 2009 virus at the signature positions was also elucidated by applying the entropy-based signature analysis to these sequences. They were found mostly to be characteristic of avian species, as presented in Table 1. Notably, 8 of them changed from avian-like signatures to human-like signatures. Close examination of the residue transition at these 8 positions in Table 3 showed that PA-356, unlike the other 7 positions, retained an avian-like signature in the recent ancestral swine population and changed to a human-like signature only in pandemic (H1N1) 2009. This finding suggests that PA-356 may be related to host-restriction factors from swine to human species. Similarly, all ribonucleoprotein positions were scanned for the same transitioning pattern as in PA-356, i.e., a retained avian-like residue in the recent ancestral swine population and a change to the human residue in pandemic (H1N1) 2009 viruses. Table 4 lists them all. Although 1 of the positions, PB1- 216, was not dominated by the residue S as we would have expected, it exhibited a mixture of 2 residues involving a transition from avian to human viruses. In summary, Table 4 provides a list of candidate host-restriction factors that we believe are important to adaptive mutation of influenza A viruses among the 3 host species. Continuous monitoring of these signatures in nonhuman species will help in influenza surveillance and in evaluating the likelihood of further adaptation to humans.

## Supplementary Material

Appendix Table Recent ancestral swine influenza A viruses of pandemic (H1N1) 2009 viruses and their accession numbers for PB2, PB1, PA, and NP protein sequences*
