# Supplementary material for: Genomic Signatures of Influenza A Pandemic (H1N1) 2009 Virus
Source: Emerg Infect Dis. 2009 Dec;15(12):1897–903. doi: 10.3201/eid1512.090845 (PMC3375880; doi:10.3201/eid1512.090845)
Supplement: Appendix Table — Recent ancestral swine influenza A viruses of pandemic (H1N1) 2009 viruses and their accession numbers for PB2, PB1, PA, and NP protein sequences* [file 09-0845_appT-s1.pdf]

Publisher: CDC; Journal: Emerging Infectious Diseases
Article Type: Research; Volume: 15; Issue: 12; Year: 2009; Article ID: 09-0845
DOI: 10.3201/eid1512.090845; TOC Head: Research

Appendix Table. Recent ancestral swine influenza A viruses of pandemic (H1N1) 2009 viruses and their accession numbers for PB2, PB1, PA, and NP protein sequences*

Strain	Subtype	PB2	PB1	PA	NP
A/swine/Minnesota/593/99	H3N2	AAG01793	AAG01787	AAG01792	AAG01789
A/swine/Hong Kong/NS623/2002	H1N2	ACR84054	ACR84039	ACR84021	ACR83991
A/swine/Hong Kong/78/2003	H1N2	ACR84047	NA	ACR84014	ACR83984
A/swine/North Carolina/2003	H3N2	ABQ41895	ABQ41896	ABQ41897	ABQ41899
A/swine/MI/PU243/2004	H3N1	ABA27429	ABA27430	ABA27431	ABA27433
A/swine/IN/PU542/2004	H3N1	ABA27437	ABA27438	ABA27439	ABA27441
A/swine/Hong Kong/915/2004	H1N2	ACR84042	ACR84025	ACR84009	ACR83979
A/swine/Alberta/14722/2005	H3N2	ABF18003	ABF18009	ABF17997	ABF17979
A/swine/Manitoba/12707/2005	H3N2	ABF18005	ABF18011	ABF17999	ABF17981
A/swine/Ontario/33853/2005	H3N2	ABF18006	ABF18012	ABF18000	ABF17982
A/swine/Hong Kong/1562/2005	H1N2	ACR84050	ACR84034	ACR84017	ACR83987
A/swine/Missouri/4296424/2006	H2N3	ABY40446	ABY40445	NA	ABY40441
A/swine/Guangxi/13/2006	H1N2	ABR87897	ABR87896	ABR87895	ABR87894
A/swine/Hong Kong/1110/2006	H1N2	ACR84055	ACR84040	ACR84022	ACR83992
A/swine/Shanghai/1/2007	H1N2	ACI48761	ACI48762	ACI48768	ACI48765
A/swine/OH/511445/2007	H1N1	ACH69549	ACH69550	ACH69552	ACH69553
A/swine/Korea/C13/2008	H5N2	ACJ53898	NA	ACJ53892	ACJ53886
A/swine/Hong Kong/294/2009	H1N2	ACR84051	ACR84035	ACR84018	ACR83988

*PB, RNA polymerase B; PA, RNA polymerase; NP, nucleoprotein; NA, excluded. Swine viruses from sister lineages of pandemic (H1N1) 2009 viruses according to supplementary Figure 2 of Smith et al. (*6*). We consider recent ancestral swine viruses phylogenetically neighboring to the pandemic 2009 strains, in particular for PB2 and PA genes they are clustered together with recent avian strains because that the pandemic H1N1 viruses were reportedly originated from avian virus. Note 1 strain, A/swine/Missouri/4296424/06(H2N3), whose PA sequence was not found anywhere near the other 17 recent swine strains of interest. Two PB1 sequences of A/swine/Hong Kong/78/2003(H1N2) and A/swine/Korea/C13/2008(H5N2) were found distantly located from the other 16 recent swine PB1 sequences. Because they may have acquired certain genetic diversity through other evolutionary paths and thus could bias the discussion of residue transitions between pandemic (H1N1) 2009 viruses and their immediate ancestral swine viruses, these 3 sequences were excluded (labeled NA) from amino acid statistics here. Regardless, the inclusion of these 3 sequences does not change the conclusion drawn from Tables 3 and 4 in the text.
